# Targeted Expansion of Tissue-Resident CD8^+^ T Cells to Boost Cellular Immunity in the Skin

**DOI:** 10.1016/j.celrep.2019.10.126

**Published:** 2019-12-03

**Authors:** Samuel J. Hobbs, Jeffrey C. Nolz

**Affiliations:** 1 Department of Molecular Microbiology and Immunology, Oregon Health & Science University, Portland, OR 97239, USA; 2 Department of Cell, Developmental and Cancer Biology, Oregon Health & Science University, Portland, OR 97239, USA; 3 Department of Radiation Medicine, Oregon Health & Science University, Portland, OR 97239, USA; 4 Lead Contact

## Abstract

Tissue-resident memory (T_RM_) CD8^+^ T cells are positioned within environmental barrier tissues to provide a first line of defense against pathogen entry, but whether these specialized T cell populations can be readily boosted to increase protective immunity is ill defined. Here, we demonstrate that repeated activation of rare, endogenous T_RM_ CD8^+^ T cells, using only topical application of antigenic peptide causes delayed-type hypersensitivity and increases the number of antigen-specific T_RM_ CD8^+^ T cells, specifically in the challenged skin by ~15-fold. Expanded T_rm_ CD8^+^ T cells in the skin are derived from memory T cells recruited out of the circulation that became CD69^+^ tissue residents following a local antigen encounter. Notably, recruited circulating memory CD8^+^ T cells of a different antigen specificity could be coerced to become tissue resident using a dual-peptide challenge strategy. Expanded T_RM_ CD8^+^ T cells significantly increase anti-viral protection, suggesting that this approach could be used to rapidly boost tissue-specific cellular immunity.

## In Brief

Tissue-resident memory (T_RM_) T cells provide a first line of host defense against pathogen invasion at environmental barrier tissues. Here, Hobbs and Nolz describe a mechanism to rapidly expand the number of antigen-specific T_RM_ CD8^+^ T cells in the skin, using topical application of antigenic peptide to boost localized protective immunity.

## Graphical Abstract

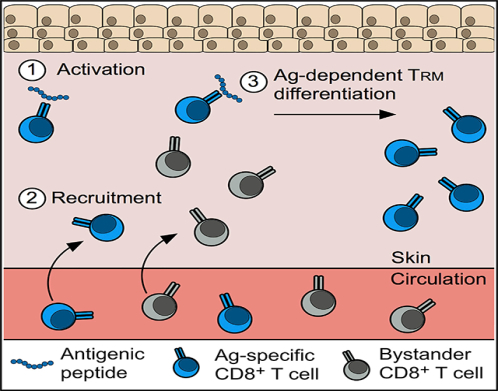

## INTRODUCTION

Cellular immunity is largely mediated by CD4^+^ and CD8^+^ T cells and requires direct recognition of “non-self” peptides presented on major histocompatibility complexes (MHCs). Because many intracellular infections occur within non-lymphoid tissues, memory T cells must either be already positioned at the site of pathogen entry or be able to rapidly localize to inflamed tissues following re-infection. Traditionally, the goal of vaccination strategies targeting the formation of cellular immunity has been to generate large populations of circulating antigen (Ag)-specific memory T cells with booster immunizations and strong adjuvants (Gilbert, 2012; Slifka and Amanna, 2014). In theory, expanding the number of memory T cells in the circulation would lead to increased surveillance of peripheral tissues and responsiveness to secondary challenge. However, in human vaccination trials targeting the prevention of AIDS, tuberculosis, and malaria, the numbers of circulating memory T cells have not correlated with protection, even after successful heterologous boosting (Buchbinder et al., 2008; McNatty et al., 2000; Tameris et al., 2013). This lack of protection by circulating memory T cells has generated a strong interest in developing vaccines that seed tissue-resident memory (T_RM_) T cells at sites of pathogen entry.

Although the factors governing the differentiation of T_RM_ cells are not completely understood, recruitment of effector T cells into peripheral tissues can be sufficient to generate a T_RM_ population (Casey et al., 2012; Mackay et al., 2012). Thus, one approach to seed T_RM_ cells within a target tissue is to prime a T cell response and recruit effector T cells into the tissue microenvironment by delivering recombinant chemokines or other non-specific inflammatory agents. Recent studies have reported that T_RM_ cells generated using this “prime and pull” approach are highly protective against both infections and tumors (Gálvez-Cancino et al., 2018; Mackay et al., 2012; Shin and Iwasaki, 2012). However, the chemokines used in the recruitment phase only recruit effector (and not memory) CD8^+^ T cells; as a result, this technique only allows a short time frame in which seeding of T_RM_ cells can occur and cannot be used to transfer of monoclonal T cell receptor transgenic (TCR-tg) T cells may not accurately reflect the same trafficking and localization boost existing T_RM_ populations (Shin and Iwasaki, 2012). Further, the large population of effector and memory cells resulting from the patterns of the relatively rare, polyclonal endogenous Ag-specific CD8^+^ T cell repertoire (Badovinac et al., 2007). Here, we show that topical application of antigenic peptide to skin harboring endogenous T_RM_ CD8^+^ T cells causes inflammation and locally expands the Ag-specific (but not bystander) T_RM_ population by recruiting new T_RM_ precursors from the circulation. This mechanism of T_RM_ expansion significantly improved protective immunity in the skin, suggesting its potential utility as a tissue- and Ag-specific vaccine boosting strategy.

## RESULTS

### Viral Skin Infection Generates Protective Circulating and Tissue-Resident Memory T Cells

**Figure 1.**
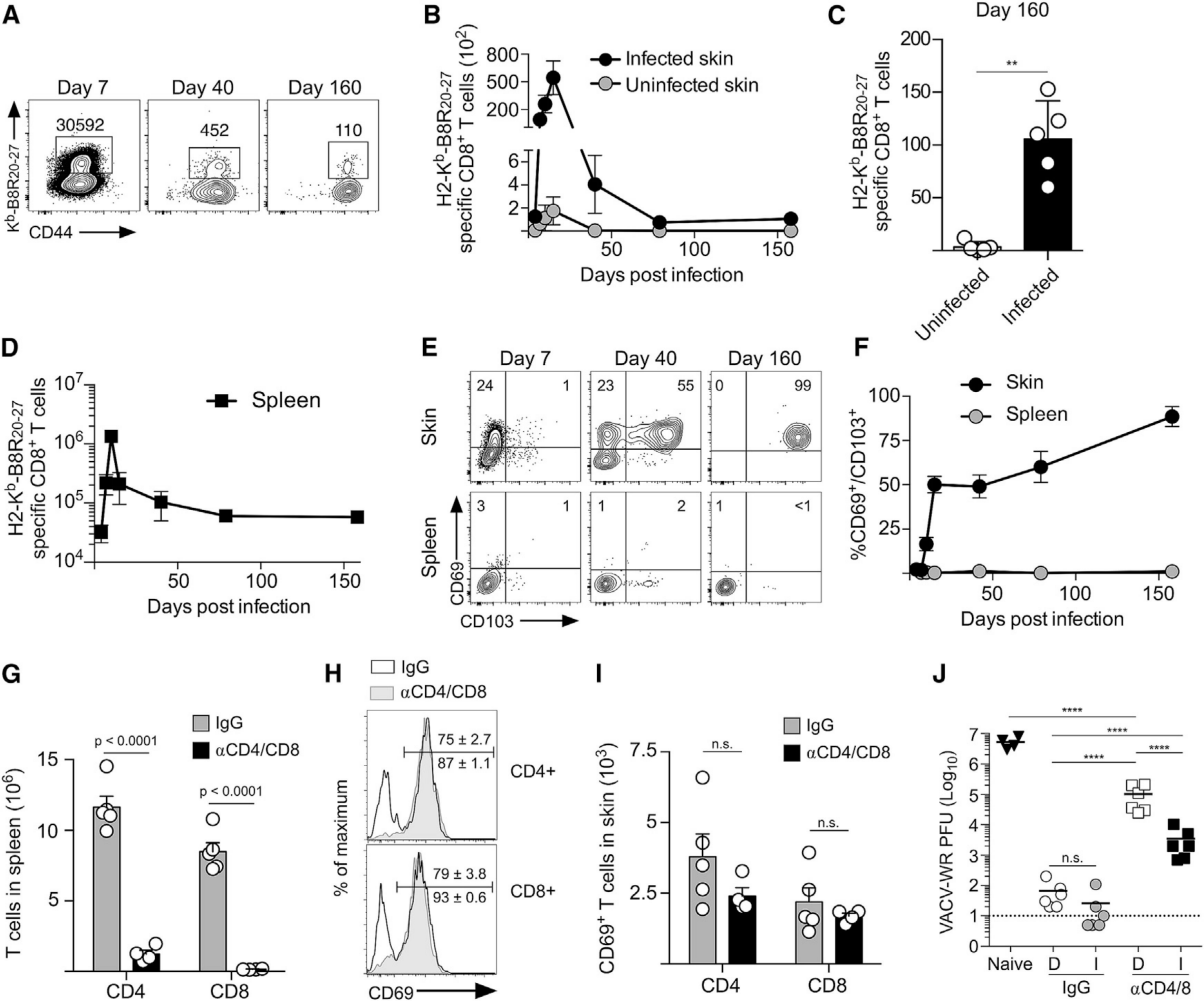
VACV Skin Infection Generates Protective Circulating and Tissue-Resident Memory T Cell Populations (A) Mice were infected on the left ear skin with VACV, and B8R-specific CD8^+^ T cells from infected skin were identified at the indicated times post-infection by flow cytometry. (B) Quantification of (A). (C) Quantification of the final time point in (B). (D) Quantification of B8R-specific CD8^+^ T cells in the spleen. (E) CD103 and CD69 expression by B8R-specific CD8^+^ T cells. (F) Quantification of (E). (G-I) Mice were infected as in (A) and received IgG or CD4/CD8 depleting antibodies on day 35 post-infection. (G) Number of CD4^+^ and CD8^+^ T cells in the spleen. (H) CD69 expression by CD4^+^ and CD8^+^ T cells in the skin. (I) Number of CD69^+^ CD4^+^ and CD8^+^ T cells in the skin. (J) Mice were treated as in (G) and challenged on left and right ear skin with VACV-WR. Viral load was quantified day 3 post-infection. Dashed line indicates limit of detection. D, distal skin; I, immunized skin. Statistical significance was determined using a paired t test (C), unpaired t test (G and I), or one-way ANOVA with Tukey’s multiple comparison test (J). Error bars represent SEM. Data in (A)–(I) are representative of at least 2 independent experiments (n = 3–5). Data in (J) are pooled from 2 independent experiments (n = 3).

Skin infection with poxvirus vectors has become an attractive and widely used vaccine approach (Pastoret and Vanderplasschen, 2003). Using a procedure similar to the smallpox immunization strategy (Hickman et al., 2013), we infected the left ear skin of naive B6 mice with attenuated, thymidine kinase deficient *(tk–)* vaccinia virus (VACV) (Buller et al., 1985) and analyzed the accumulation of CD8^+^ T cells in the skin that were specific for the immunodominant epitope of VACV (H2-K^b^-B8R_20–27_). B8R-specific CD8^+^ T cells trafficked into the infected skin between days 7 and 15 post-infection, and a stable population of 50–150 B8R-specific memory CD8^+^ T cells formed in the previously infected skin ~80 days after infection (Figures 1A–1C). B8R-specific CD8^+^ T cells that remained in the skin expressed the canonical T_RM_ markers CD69 and CD103, whereas memory B8R-specific CD8^+^ T cells in the spleen did not (Figures 1D–1F). Together, these data demonstrate that VACV skin immunization generates Ag-specific memory CD8^+^ T cells in both the circulation and in the skin.

VACV infection generates robust humoral and cellular immunity that accelerates clearance of a secondary infection (Hammarlund et al., 2003). To quantify the amount of protection provided by these arms of adaptive immunity, we treated mice that were immunized only on the left ear skin with control (immunoglobulin G; IgG) or anti-CD4/CD8 depleting antibodies (αCD4/8), which eliminated nearly all T cells from the spleen (Figure 1G) and CD69^−^ cells from the skin but did not deplete CD69^+^ T cells at the vaccination site (Figures 1H and 1I). Both the left and right ear skin were then infected with the more virulent parent strain of VACV (Western Reserve; VACV-WR). IgG-treated animals prevented VACV infection at the site of immunization as well as in distal unimmunized skin (Figure 1J, IgG), demonstrating that the combination of circulating memory T cells and antibodies can be sufficient to rapidly control viral skin infection. However, when circulating memory T cells were eliminated, viral titers were reduced in immunized skin ~50-fold compared to those in distal skin (Figure 1J, αCD4/8). These data demonstrate that VACV skin infection generates highly protective adaptive immunity, but endgogenous T_RM_ cells provide site-specific protection even within the context of functional humoral immunity.

### Activation of T_RM_ CD8^+^ T Cells Causes Delayed Type Hypersensitivity and Local Accumulation of Ag-Specific T_RM_ CD8^+^ T Cells

**Figure 2.**
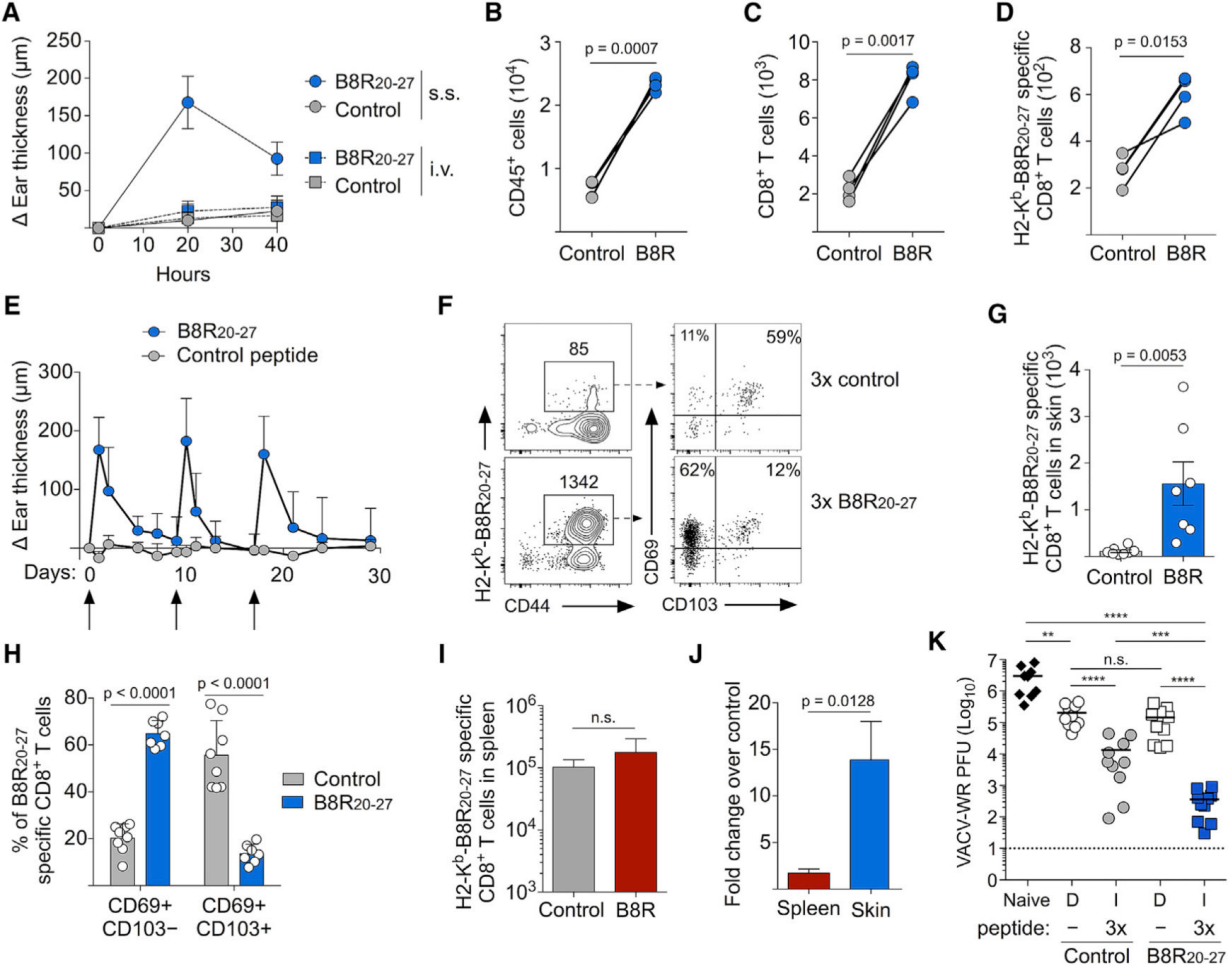
Topical Application of Cognate Peptide Activates T_RM_ CD8^+^ T Cells and Boosts the Quantity of Antigen-Specific T_RM_ CD8^+^ T Cells in the Skin (A) Mice were infected with VACV i.v. or on the left ear skin by scarification (s.s.; see Figure S1). On day 40 post-infection, B8R_20–27_ or control peptide (NP_396–404_ of LCMV) was applied to the left ear skin, and change in ear thickness was measured. (B–D) Mice were infected by scarification as in (A), and 40 h after peptide application, the numbers of CD45^+^ (B), CD8^+^ (C), and B8R-specific CD8^+^ (D) T cells in the skin were determined by flow cytometry. (E) Mice were infected on the left and right ear skin with VACV. On day 35 post-infection, skin was challenged with B8R_20–27_ or control peptide, and swelling was monitored. Once swelling had subsided, mice were re-challenged two more consecutive times (arrows indicate peptide challenge). (F) Representative flow cytometry plots depicting B8R-specific CD8^+^ T cells and expression of CD69 and CD103 10 days after the final peptide challenge (see Figure S2). (G) Quantification of the number of B8R-specific CD8^+^ T cells in (F). (H) Quantification of CD103 and CD69 expression by B8R-specific CD8^+^ T cells identified in (F). (I) Number of B8R-specific CD8^+^ T cells in the spleen of mice challenged with control peptide or B8R_20–27_. (J) Fold increase in B8R-specific CD8^+^ T cells in spleen or skin after B8R_20–27_ challenge. (K) Mice were treated as in (E) and were given anti-CD4/CD8 depleting antibodies 10 days after the last peptide challenge. Mice were then infected on both ear skins with VACV-WR, and viral load was quantified day 3 post-infection. Dashed line indicates limit of detection. D, distal skin; I, immunized skin. Statistical significance was determined using a paired t test (B–D), unpaired t test (G–J), or one-way ANOVA with Tukey’s multiple comparison test (K). Error bars represent SEM. Data in (A)–(J) are representative of at least 3 independent experiments (n = 3–8). Data in (K) are pooled from 2 independent experiments (n = 4–5).

Topical application of antigenic peptide to T_RM_-containing skin causes delayed type hypersensitivity (DTH), characterized by tissue swelling and the recruitment of circulating lymphocytes (Ariotti et al., 2014; Gaide et al., 2015; Khan et al., 2016; Schenkel et al., 2013, 2014). Because only 50–150 B8R-specific T_RM_ CD8^+^ T cells formed in the skin after VACV infection, we next tested whether this small population could also initiate a DTH response. We infected the left and right ear skin with *tk–* VACV and, at 35 days post-infection, topically applied a DMSO/acetone solution containing B8R_20–27_ to the left ear skin and control peptide to the contralateral ear skin. B8R-challenged skin became inflamed (Figure 2A) and rapidly accumulated CD45^+^ leukocytes, CD8^+^ T cells, and B8R-specific CD8^+^ T cells (Figures 2B–2D). Peptide dissolved in a skin-penetrating DMSO/acetone solution was required, as leukocytes did not accumulate in skin challenged with B8R_20–27_ suspended in an olive oil emulsion (Figure S1A). T_RM_ CD8^+^ T cells were also necessary for this DTH reaction, because the skin of mice infected intravenously (i.v.) with VACV, which generated circulating B8R-specific memory CD8^+^ T cells but limited numbers of T_RM_ CD8^+^ T cells in the skin (Figures S1B–S1D), did not become inflamed after B8R_20–27_ challenge (Figure 2A). To determine the duration and durability of the DTH response, we allowed inflammation to subside (~10 days) and then re-challenged the skin with B8R_20–27_ two additional times. The inflammatory response was a similar magnitude and duration following each challenge (Figure 2E), demonstrating that Ag-specific T_RM_ CD8^+^ T cells remain highly functional after repeated activation.

We next determined whether repeated activation caused any changes to the B8R-specific T_RM_ CD8^+^ T cell population in the challenged skin. Following resolution of the final episode of inflammation, the number of B8R-specific T_RM_ CD8^+^ T cells increased significantly in B8R-stimulated skin compared to that in control skin (Figures 2F and 2G). B8R-specific T_RM_ CD8^+^ T cells in skin challenged with control peptide remained mostly CD69^+^/CD103^+^, whereas the expanded B8R-specific T_RM_ population became largely CD69^+^/CD103^−^ (Figures 2F and 2H), which we have recently shown to be the dominant T_RM_ CD8^+^ T cell population that forms following secondary VACV skin infection (Osborn et al., 2019). Notably, T_RM_ CD8^+^ T cells that were not B8R specific did not expand and remained CD69^+^/CD103^+^ (Figures S2A–S2C). B8R-specific CD8^+^ T cells in the spleen were not significantly boosted by peptide challenge (Figure 2I), demonstrating that expansion was site specific and restricted to the Ag-specific T_RM_ CD8^+^ T cell population (Figure 2J). Critically, the increased number and CD69^+^/CD103^−^ phenotype of B8R-specific CD8^+^ T cells were maintained 40 days after peptide challenge (Figures S2D–S2F), demonstrating the stability of the expanded B8R-specific T_RM_ population.

Because B8R-specific T_RM_ CD8^+^ T cells expanded exclusively in the skin following three rounds of B8R_20–27_ peptide challenge, we next determined whether boosting T_RM_ CD8^+^ T cells of a single Ag specificity would improve protection compared to the primary T_RM_ population. We administered 3 rounds of B8R_20–27_ or control peptide to VACV-immunized skin (as in Figure 2E), depleted circulating CD4^+^ and CD8^+^ T cells, and infected both distal and immunized skin with VACV-WR (as in Figure 1J). In immunized skin, boosting the number of B8R-specific T_RM_ CD8^+^ T cells increased protection by ~50-fold compared to the primary T_RM_ population (Figure 2K), whereas protection in distal skin was not affected. Together, these data demonstrate that activation of endogenous skin T_RM_ CD8^+^ T cells causes acute DTH but also generates a larger population of secondary Ag-specific T_RM_ CD8^+^ T cells that provide site-specific protective immunity.

### Circulating Memory CD8^+^ T Cells Traffic into the Skin following Local T_RM_ Activation and Accumulate in an Ag-Specific Manner

**Figure 3.**
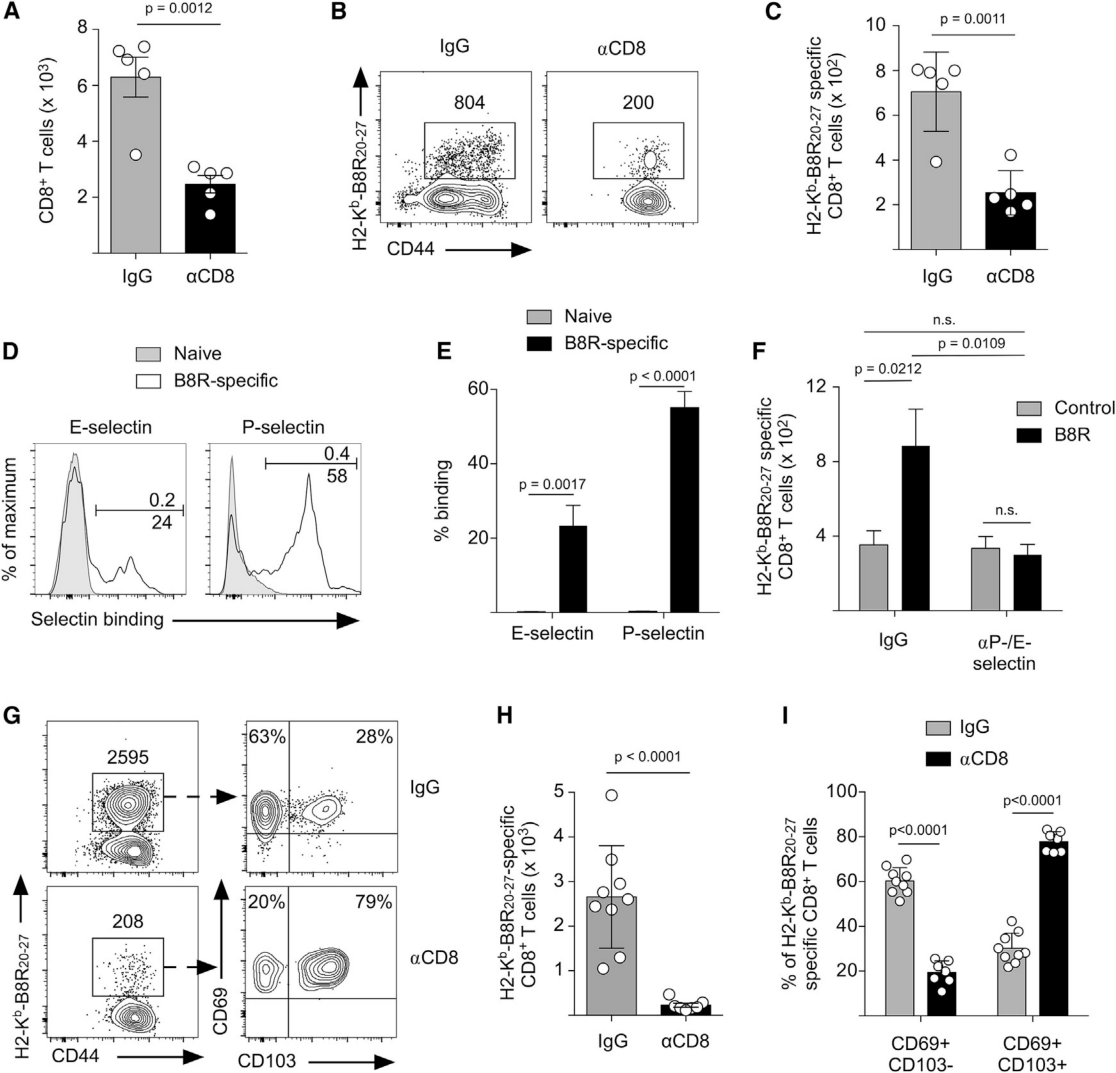
Circulating Memory CD8^+^ T Cells Are Recruited to the Site of T_RM_ Activation and Are the Source of Secondary T_rm_ CD8^+^ T Cells (A–C) Mice were infected on the left ear skin with VACV and received control IgG or CD8 depleting antibody 35 days post-infection. Mice were then challenged with B8R_20–27_, and the number of CD8^+^ T cells (A) and B8R_20–27_-specific CD8^+^ T cells (B and C) was quantified 40 h after challenge. (B) Representative flow cytometry plots depicting B8R-specific CD8^+^ T cells in the challenged skin. (C) Quantification of (B). (D) Mice were infected on the skin with VACV, and on day 35 after infection, B8R-specific CD8^+^ T cells in the blood were analyzed for expression of P- and E-selectin ligands. Naive CD8^+^ T cells (CD62L^+^/CD44^−^) were used as a negative control for selectin binding. (E) Quantification of (D). (F) Mice were infected on the skin with VACV and received control IgG or P-/E-selectin blocking antibodies before challenge with B8R_20–27_ or control peptide on day 40 post-infection. The number of B8R-specific CD8^+^ T cells in the skin 40 h after challenge was determined by flow cytometry (see Figure S3). (G) Mice were treated as in (A), except they were challenged three times with B8R_20–27_ (as in Figure 2E) and analyzed by flow cytometry 10 days after the last peptide challenge. (H) Quantification of the number of B8R-specific CD8^+^ T cells in (G). (I) Quantification of CD103 and CD69 expression by B8R-specific CD8^+^ T cells identified in (G). Statistical significance was determined using an unpaired t test. Error bars represent SEM. Data in (A)–(C) are representative of 2 independent experiments (n = 3–5). Data in (D)–(F) are representative of 3 independent experiments (n = 3–10). Data in (G)-(I) are pooled from 2 independent experiments (n = 4–5).

To determine whether the rapid accumulation of CD8^+^ T cells following T_RM_ activation was due to the recruitment of circulating memory CD8^+^ T cells, we treated mice with CD8 depleting antibody before peptide challenge, which eliminates CD8^+^ T cells from the circulation but spares T_RM_ CD8^+^ T cells in the skin (Figures 1G–1I). When circulating CD8^+^ T cells were depleted, the number of CD8^+^ T cells and B8R-specific cells did not increase after peptide challenge (Figures 3A–3C), demonstrating that the increase of CD8^+^ T cells in the skin within 40 h of peptide challenge was due to the recruitment of memory CD8^+^ T cells from the circulation. Trafficking of memory CD8^+^ T cells into inflamed skin is dependent on their ability to bind P-/E-selectin on vascular endothelium (Nolz and Harty, 2014). B8R-specific memory CD8^+^ T cells in the circulation expressed P- and E-selectin ligands (Figures 3D and 3E), and blocking P- and E-selectin prevented recruitment of CD45^+^ and CD8^+^ T cells (Figures S3A and S3B) and B8R-specific CD8^+^ T cells into the skin (Figure 3F). To determine whether recruitment of circulating Ag-specific memory CD8^+^ T cells was required for the subsequent formation of an expanded, stable secondary T_RM_ CD8^+^ T cell population, we depleted circulating CD8^+^ T cells and challenged the ear skin with 3 rounds of B8R_20–27_. As shown previously (Figures 2F–2H), B8R-specific CD8^+^ T cells in the skin of IgG-treated mice expanded and formed a CD69^+^/CD103^−^ secondary T_RM_ population (Figures 3G–3I). In contrast, mice that lacked circulating memory CD8^+^ T cells failed to accumulate B8R-specific T_RM_ CD8^+^ T cells and remained mostly CD69^+^/CD103^+^ (Figures 3G–3I). Together, these data demonstrate that the accumulation of CD69^+^/CD103^−^ Ag-specific T_RM_ CD8^+^ T cells requires the recruitment of memory CD8^+^ T cells from the circulation.

### “Recruit and Capture” Strategy to Establish Ag-Specific T_rm_ CD8^+^ T Cells in the Skin

**Figure 4.**
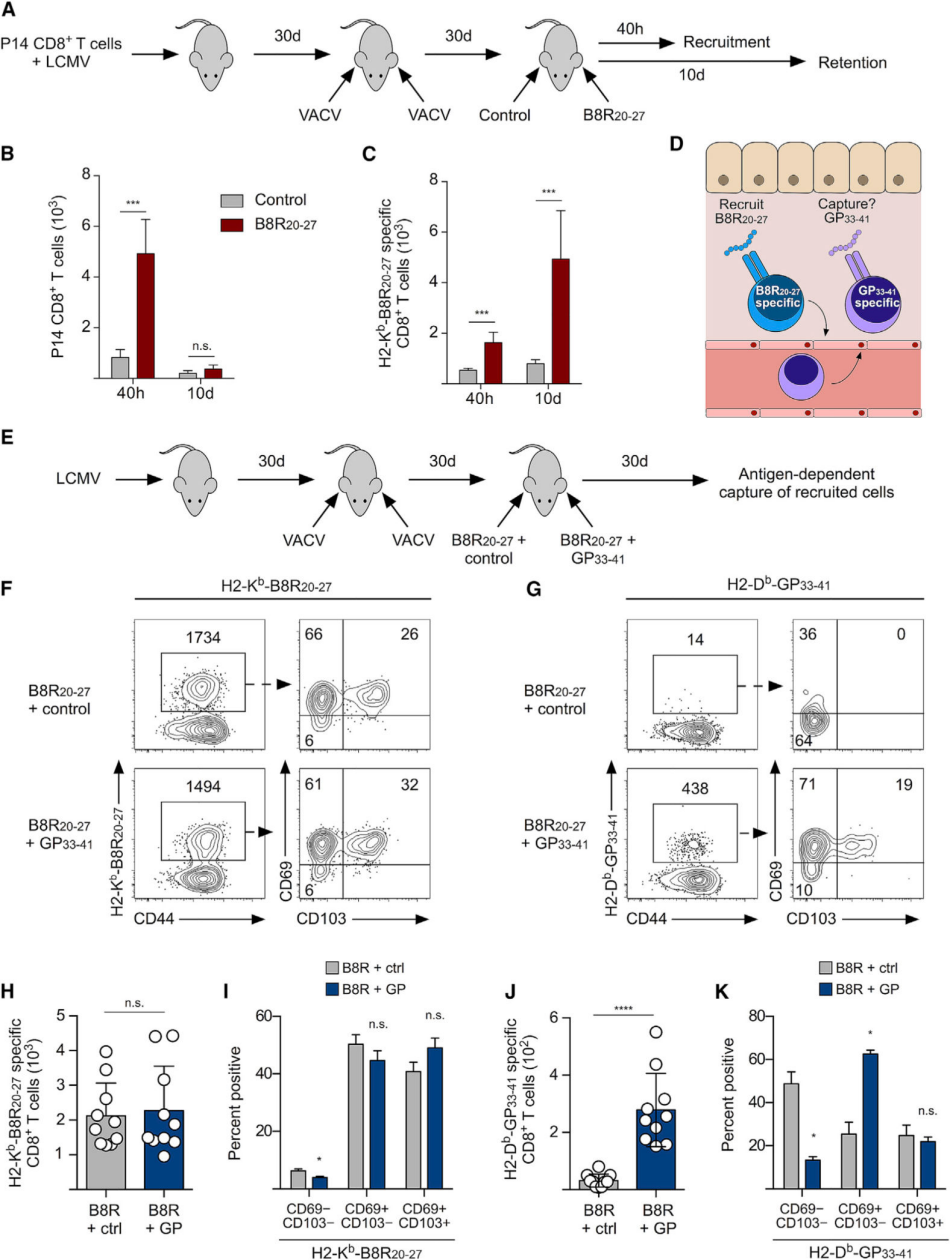
Circulating Memory CD8^+^ T Cells Recruited during DTH Form a *de novo* T_rm_ CD8^+^ T Cell Population following Local Antigen Recognition (A–C) Mice received naive P14 CD8^+^ T cells and were infected with LCMV. Mice were then infected with VACV on the left and right ear skin, followed by challenge with either control peptide (NP_396–404_) or B8R_20–27_. (A) Experimental design. (B and C) Number of P14 (B) or B8R-specific (C) CD8^+^ T cells in the skin 40 h after peptide challenge or 10 days after three rounds of peptide challenge. (D) Schematic of the “recruit and capture” strategy. (E–K) Mice were infected with LCMV and, 30 days later, were infected with VACV. Mice were then challenged three times with a mixture of B8R plus control peptide (OVA_257–264_) or B8R plus GP_33–41_, and skin was analyzed 30 days after the final peptide challenge. (E) Experimental design. (F and G) Representative flow cytometry plots depicting the number of B8R-specific (F) or GP_33–41_-specific (G) CD8^+^ T cells and their expression of CD69 and CD103. (H) Quantification of the number of B8R-specific CD8^+^ T cells in (F). (I) Quantification of CD103 and CD69 expression by B8R-specific CD8^+^ T cells identified in (F). (J) Quantification of the number of GP_33–41_-specific CD8^+^ T cells in (G) (see Figure S4). (K) Quantification of CD103 and CD69 expression by GP_33–41_-specific CD8^+^ T cells identified in (G). Statistical significance was determined using a paired t test. Error bars represent SEM. Data in (B) and (C) are representative of 2 independent experiments (n = 5). Data in (F)–(K) are representative of 3 independent experiments (n = 4–10).

It has been reported that recruitment of effector/memory CD8^+^ T cells into non-lymphoid tissues can be sufficient to generate T_RM_ CD8^+^ T cells (Beura et al., 2018; Casey et al., 2012; Davies et al., 2017; Mackay et al., 2013; Shin and Iwasaki, 2012). To test whether this occurs during a T_RM_-mediated DTH reaction, we transferred naive P14 CD8^+^ T cells and infected mice with lymphocytic choriomeningitis virus (LCMV), which generates robust circulating memory populations, but limited numbers of P14 CD8^+^ T cells can be isolated from the skin (Osborn et al., 2019). LCMV-immune mice were then infected on the ear skin with VACV to generate B8R-specific T_RM_ CD8^+^ T cells. Mice were then challenged with B8R_20–27_ or control peptide, and recruitment of P14 and B8R-specific CD8^+^ T cells was analyzed (Figure 4A). Both memory P14 and B8R-specific CD8^+^ T cells rapidly trafficked into the skin within 40 h of B8R_20–27_ challenge (Figures 4B and 4C, 40h), demonstrating that the recruitment of circulating memory CD8^+^ T cells is antigen independent. However, in skin that was challenged with B8R peptide, only B8R-specific CD8^+^ T cells were retained 10 days after the final challenge (Figures 4B and 4C, d), demonstrating that there is, essentially, no bystander T_RM_ differentiation of recruited memory CD8^+^ T cells. This finding suggested that the B8R_20–27_ peptide was responsible for both initiating recruitment and the subsequent accumulation of B8R-specific memory CD8^+^ T cells in the skin. Thus, we next tested whether a new T_RM_ population could be established by recruiting circulating memory CD8^+^ T cells with B8R_20–27_ and then “capturing” memory CD8^+^ T cells of a different Ag specificity by including an additional antigenic peptide (Figure 4D). To test this, we infected LCMV-immune mice on the left and right ear skin with VACV and, 30 days later, challenged the left ear skin with B8R_20–27_/GP_33–41_ and the right ear skin with B8R_20–27_/control peptide 3 times (Figure 4E). Thirty days after the final peptide challenge, B8R-specific CD8^+^ T cells accumulated at both sites equally, but significantly more GP_33–41_-specific CD8^+^ T cells were retained in the B8R_20–27_/GP_33–41_-challenged skin compared to those in the B8R_20–27_/control-challenged skin (Figures 4F–4K). B8R-specific CD8^+^ T cells were largely CD69^+^/CD103^−^ at both sites, while the majority of GP_33–41_-specific CD8^+^ T cells were CD69^−^/CD103^−^ in B8R_20–27_/control-challenged skin and mostly CD69^+^/CD103^−^ in B8R_20–27_/GP_33–41_-challenged skin (Figures 4F–4K). Similar results were observed when TCR-tg P14 CD8^+^ T cells were used as the “capture” population (Figure S4). Together, these data demonstrate that activation of established T_RM_ CD8^+^ T cells is sufficient to recruit circulating memory CD8^+^ T cells, but the capture and subsequent differentiation into CD69^+^ T_RM_ require Ag recognition within the skin.

## DISCUSSION

Recently, we reported that the presence of local antigen enhances the formation of T_RM_ CD8^+^ T cells during a primary VACV skin infection (Khan et al., 2016), which agrees with our data presented here, demonstrating that antigen recognition by circulating memory CD8^+^ T cells within the skin microenvironment also controls the formation and retention of secondary T_RM_ cells. This finding contrasts with a recent study demonstrating bystander T_RM_ differentiation following herpes simplex virus 1 (HSV-1) skin infection (Park et al., 2018), suggesting that the mechanisms controlling T_RM_ differentiation may also be dictated by the nature of the inflammatory microenvironment. Most secondary T_RM_ CD8^+^ T cells do not express CD103, which is consistent with our recent observation that skin-T_RM_ CD8^+^ T cells that are derived from circulating memory cells are intrinsically unable to express CD103, but are, in fact, T_RM_ cells (Osborn et al., 2019). Interestingly, human skin contains approximately twice as many T cells as the entire vascular system, and these skin-resident T cells are nearly all CD69^+^ but heterogeneous in terms of CD103 expression (Clark, 2015). In contrast, T_RM_ populations generated in laboratory mice following a single infection are generally smaller than circulating T cell populations and nearly all CD69^+^/CD103^+^ (Gebhardt et al., 2009; Mackay et al., 2013). Our results suggest that recurring encounters with pathogens or environmental antigens may contribute to the increased size and varied composition of the CD8^+^ T cell populations observed in human skin.

In summary, we demonstrate that topical application of a single antigenic peptide boosted the number of antigen-specific T_RM_ CD8^+^ T cells in the skin and was sufficient to increase local protective immunity against a homologous infection. The secondary T_RM_ CD8^+^ T population was derived from circulating memory CD8^+^ T cells that were recruited into the skin and differentiated into a CD69^+^/CD103^−^ T_RM_ CD8^+^ T cell following local antigen encounter. Importantly, because this mechanism of expansion only relies on stable circulating memory T cell populations, boosting T_RM_ populations within the skin could occur at any time after successful immunization. Additionally, because all circulating memory CD8^+^ T cells are recruited to the site of challenge, *de novo* T_RM_ populations can be generated by including additional peptides that will capture recruited memory CD8^+^ T cells. Thus, our study demonstrates that topical application of antigenic peptides may offer a cheap and simple strategy to boost protective T_RM_ CD8^+^ T cell populations at environmental barrier tissues such as the skin.

## STAR★METHODS

### LEAD CONTACT AND MATERIALS AVAILABILITY

Further information and requests for resources and reagents should be directed to and will be fulfilled by the lead contact, Jeffrey Nolz (nolz@ohsu.edu). This study did not generate new unique reagents.

### EXPERIMENTAL MODEL AND SUBJECT DETAILS

#### Mice

C57BL/6N mice (6–10 weeks of age, female) were from Charles River/NCI. For adoptive transfers, 2.5 × 10^4^ P14 CD8^+^Thy1.1^+^ T cells (Pircher et al., 1989) were injected i.v. in 200 μL of PBS. All animal experiments were approved by the OHSU Institutional Animal Care and Use Committee.

### METHOD DETAILS

#### Infections

LCMV-Armstrong infections (2 × 10^5^ pfu) were performed by i.p. injection in a volume of 200 μl. VACV-WR, *tk*- VACV, and VACV-GP_33–41_ were maintained by propogation in BSC-40 cells as previously described (Wyatt et al., 2017). VACV skin infections were performed on anesthetized mice by placing 10^7^ pfu of virus (in 10 μL of PBS) on the ventral side of the ear pinna, and then poking the virus-coated skin 25 times with a 27-gauge needle. VACV titers from infected skin was determined using standard plaque assays as described previously (Khan et al., 2016). Briefly, infected ears were removed and homogenized in 1 mL of RPMI containing 1% FBS. Homogenates were subjected to three rounds of freeze-thaw and serial dilutions were inoculated on BSC-40 cells in a 12 well plate that was then covered with 1% Seakem agarose. Plaques were visualized three days later after overnight incubation with neutral red dye. All infectious agents were approved by the OHSU Institutional Biosafety Committee.

#### Topical peptide challenge

B8R_20–27_, GP_33–41_, NP_396–404_, and OVA_257–264_ peptides were dissolved in 20–40 μl of 4:1 acetone/DMSO or olive oil/H_2_O. The DMSO/acetone formulation was chosen to enhance the penetration of the peptides and to aid in covering the entire surface area of the ear skin. The peptide solution was applied to anesthetized mice on the dorsal and ventral side of previously infected ears using a pipette tip. Ear pinna thickness was measured with a dial micrometer (Ames).

#### Leukocyte isolation from skin

Skin tissue was incubated for 1.5 h at 37°C with 1 mL HBSS (GIBCO) containing CaCl_2_ and MgCl_2_ supplemented with 125 U/ml collagenase II (Invitrogen) and 60 U/ml DNase-I (Sigma-Aldrich). Leukocytes were purified from whole-tissue suspensions by resuspending the cells in 10 mL of 35% Percoll (GE Healthcare)/HBSS followed by centrifugation at 500 g for 10 minutes at room temperature with no brake (Osborn et al., 2017). Cell numbers in skin were quantified by flow cytometry without any bead standardization.

#### Antibodies

Depleting antibodies (100–200 μg) targeting CD4 (GK1.5) or CD8α (2.43) were delivered i.p. and CD8β (YTS156.77) and CD4 (RM4–5) fluorescent antibodies were used to confirm depletion five days after antibody administration. P/E-selectin (RB40/9A9) blocking antibodies (200 μg) were administered 18 hours before and at the time of peptide challenge.

#### Cell staining and flow cytometry

Staining for surface antigens was performed in PBS/1% fetal bovine serum for 15 minutes at 4°C. For tetramer binding, cells were incubated for 45 minutes at room temp. P/E-selectin binding was determined by incubating cells with P- or E-selectin human IgG Fc chimeric proteins (R&D Systems) for 30 minutes in 1% FBS/Dulbecco’s PBS containing Ca^2+^ and Mg^2+^ (GIBCO) at room temperature. Selectin binding was detected using anti-human IgG Fc phycoerythrin (eBioscience). Cells were then stained with fluorescent antibodies as described above. Data was acquired using either a BD LSRII Flow Cytometer in the OHSU Flow Cytometry Core Facility. Flow cytometry data was analyzed using FlowJo software, version 9.9 or 10.

### QUANTIFICATION AND STATISTICAL ANALYSIS

Statistical tests and experimental details for each experiment are stated in the figure legend. Statistical tests were performed using Prism software (version 6.0; GraphPad Software). * p < 0.05, **p < 0.01, ***p < 0.001, ****p < 0.0001.

### DATA AND CODE AVAILABILITY

No new datasets or code were generated in this paper.

## Supplementary Material

1

2

## Figures and Tables

**KEY RESOURCES TABLE:** 

REAGENT or RESOURCE	SOURCE	IDENTIFIER
Antibodies		
CD3ε	Tonbo Biosciences	145–2C11
CD4	BioLegend	RM4–5; AB_312719
CD8α	BioLegend	53–6.7; AB_893423
CD8β	BioLegend	YTS156.7.7; AB_2260149
CD44	BioLegend	IM7; AB_493683
CD103	BioLegend	2E7: AB_1133989
CD69	BioLegend	H1.2F3; AB_313109
Thy1.1	BioLegend	OX-7; AB_1595487
CD45.2	BioLegend	104; AB_1186098
CD62L	BioLegend	MEL-14; AB_313095
CD4 (depleting)	BioXCell	GK1.5; AB_1107636
CD8α (depleting)	BioXCell	2.43; AB_1125541
H2-K^b^-B8R_20–27_ tetramer	NIH Tetramer Core	N/A
H2-D^b^-GP_33–41_ tetramer	Dr. John Harty, University of Iowa	N/A
P-selectin blocking antibody	BD Pharmigen	RB40
E-selectin blocking antibody	BioXCell	9A9; AB_2687816
Bacterial and Virus Strains		
Lymphocytic Choriomeningitis Virus (Armstrong)	Dr. John Harty, University of Iowa	N/A
Vaccinia virus (*tk*-)	Dr. Ann Hill, Oregon Health & Science University	N/A
Vaccinia-GP_33–41_	Dr. John Harty, University of Iowa	N/A
Vaccinia virus Western Reserve	BEI resources	NR-56
Chemicals, Peptides, and Recombinant Proteins		
B8R_20–27_ (TSYKFESV)	Biosynthesis	N/A
GP_33–41_ (KAVYNFATC)	Biosynthesis	N/A
NP_396–404_ (FQPQNGQFI)	Biosynthesis	N/A
OVA_257–264_ (SIINFEKL)	Biosynthesis	N/A
P-selectin human IgG Fc chimeric protein	R & D systems	737-PS
E-selectin human IgG Fc chimeric protein	R & D systems	575-ES
Experimental Models: Organisms/Strains		
C57/B6	Charles River	027
P14	Dr. John Harty, University of Iowa	004694
Software and Algorithms		
Prism	GraphPad	https://www.graphpad.com/scientific-software/prism/
Flowjo (Version 9.9 or 10.5)	Becton Dickinson	https://www.flowjo.com/solutions/flowjo/downloads/

## Highlights

- Topical application of peptide boosts antigen-specific T_RM_ CD8^+^ T cells in the skin
- Expanded T_RM_ T cells enhance site-specific protective immunity
- Expanded T_RM_ T cells are derived from circulating memory CD8^+^ T cells
- Recruited memory T cells become CD69^+^ T_RM_ following local antigen encounter
