# Supplementary material for: Targeted Expansion of Tissue-Resident CD8+ T Cells to Boost Cellular Immunity in the Skin
Source: Cell Rep. Author manuscript; Available in PMC 2019 Dec 16. (PMC6914228; doi:10.1016/j.celrep.2019.10.126)
Supplement: 1 [file NIHMS1545827-supplement-1.pdf]

Cell Reports, Volume *29*

Supplemental Information

Targeted Expansion of Tissue-Resident $CD8^+$ T Cells to Boost Cellular Immunity in the Skin

Samuel J. Hobbs and Jeffrey C. Nolz

Supplemental Figure 1

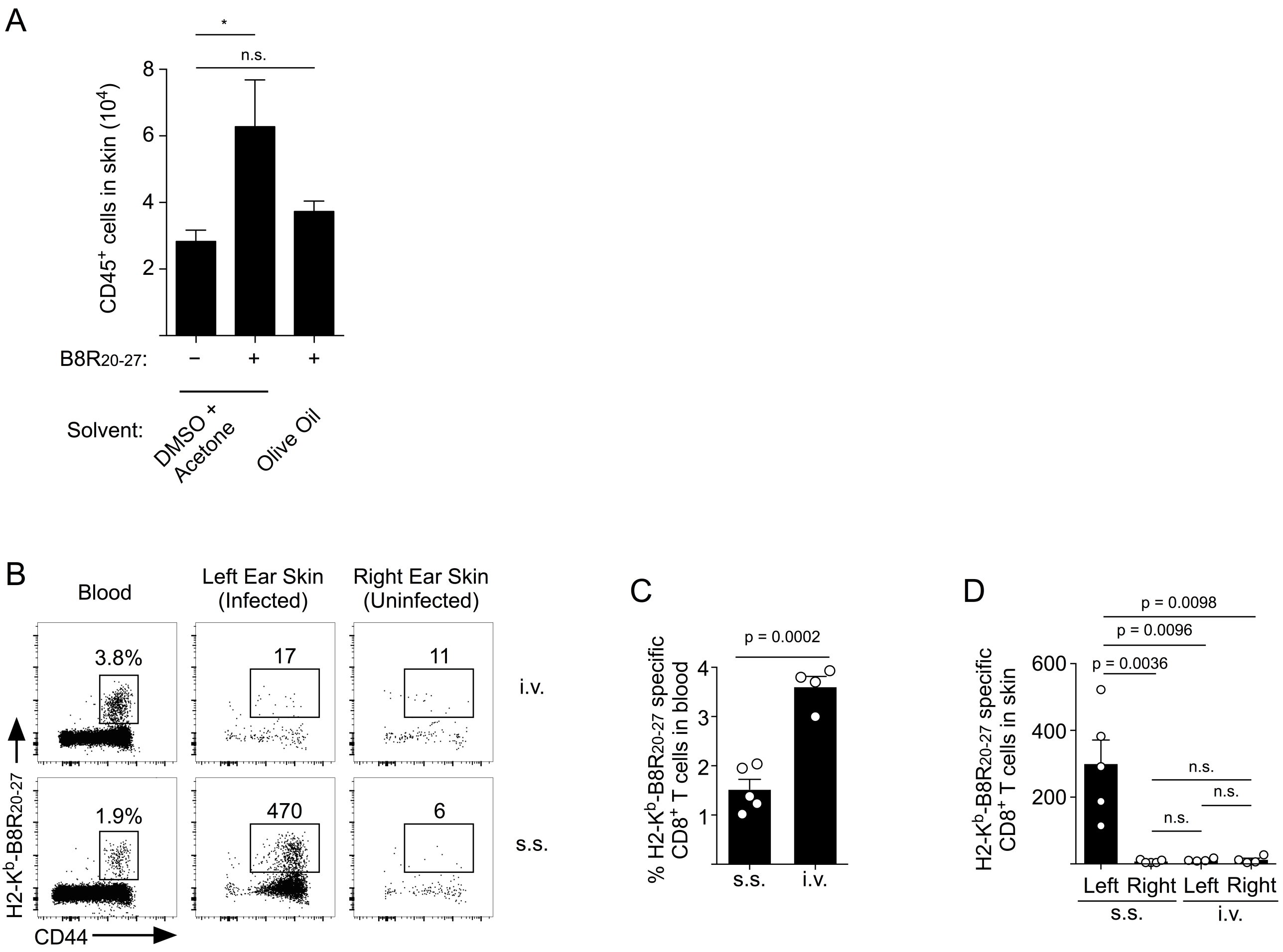

Supplemental Figure 1: DMSO is required to deliver peptide to skin-T_{RM} $CD8^+$ T cells and skin infection is required to generate functional VACV-specific skin-T_{RM} $CD8^+$ T cells (Related to Figure 2). (A) Mice were infected on the left ear skin with VACV and 30 days later were challenged with $B8R_{20\text{-}27}$ in DMSO + acetone or an olive oil emulsion and the number of $CD45^+$ cells in the challenged skin was quantified 40 hours after challenge. (B) Mice were infected with VACV either i.v. or on the left ear skin by scarification (s.s.). On day 35 post infection, B8R-specific $CD8^+$ T cells were identified in the blood and skin by flow cytometry. (C) Quantification of the frequency of B8R-specific $CD8^+$ T cells in the blood. (D) Quantification of the number of B8R-specific $CD8^+$ T cells in the left (infected) and right (uninfected) ear skin of mice infected i.v. or by scarification. Statistical significance was determined using an unpaired t-test. Data are represented as mean ± SEM. Data are representative of two independent experiments (n = 3-5).

Supplemental Figure 2

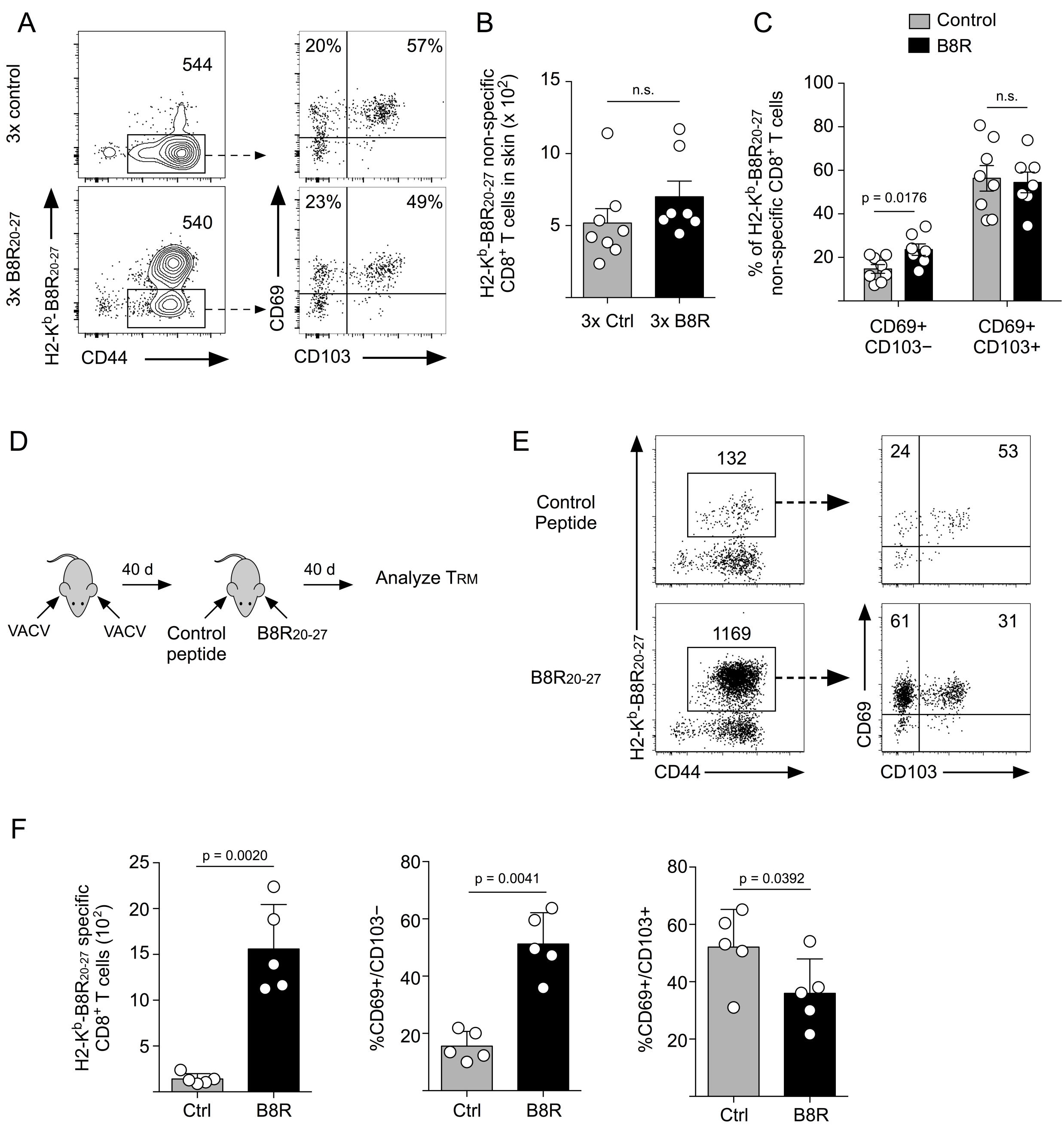

Supplemental Figure 2: Antigen non-specific CD8⁺ T cells do not accumulate in challenged skin and boosted antigen-specific T_{RM} CD8⁺ T cells are stably retained (Related to Figure 2). (A-C) Mice were infected on the left and right ear skin with VACV. On day 35 post-infection, skin was challenged with $B8R_{20\text{-}27}$ or control peptide and skin swelling was monitored. Mice were re-challenged twice more when swelling had subsided (as in Fig. 2E) and the B8R-non-specific T_{RM} $CD8^+$ T cells were analyzed 10 days after the final peptide challenge. (A) Representative flow cytometry plots depicting B8R-non-specific $CD8^+$ T cells and their expression of CD103 and CD69 (same data as in Figure 2F-H). (B) Quantification of the number of non-B8R-specific $CD8^+$ T cells in (A). (C) Quantification of CD103 and CD69 expression by non-B8R-specific $CD8^+$ T cells in (A). (D) Experimental setup to test whether the boosted B8R-specific T_{RM} population is maintained 40 days after a single round of peptide challenge. Mice were infected on the left and right ear skin with VACV and 40 days later challenged with control peptide ($NP_{396\text{-}404}$) on the right ear skin or $B8R_{20\text{-}27}$ on the left ear skin. (E) Mice were treated as in D and the number of B8R-specific T_{RM} $CD8^+$ T cells and their expression of CD103 and CD69 40 days after peptide challenge was determined by flow cytometry. (F) Quantification of E. Statistical significance was determined using an unpaired (B,C) or paired (F) t-test. Data are represented as mean ± SEM. Data are representative of at least two independent experiments (n = 3-8).

Supplemental Figure 3

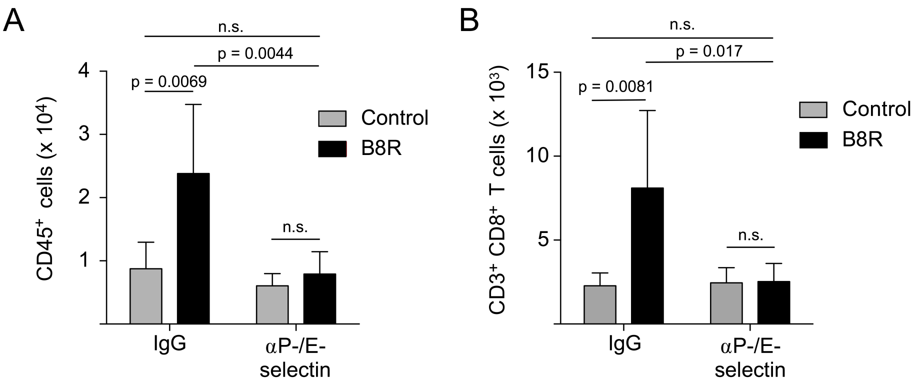

Supplemental Figure 3: Cellular recruitment associated with T_{RM}-mediated DTH is dependent on P- and E-selectin (Related to Figure 3). Mice were infected on the left and right ear skin with VACV and 35 days after infection were given control IgG or anti-P- and anti-E-selectin blocking antibodies (as in Fig. 3F). Mice were then challenged on the left ear skin with $B8R_{20-27}$ and the right ear skin with control peptide and the number of $CD45^+$ cells (A), and $CD8^+$ T cells (B) was determined by flow cytometry. Statistical significance was determined using an unpaired t-test. Data are represented as mean ± SEM. Data are representative of 3 independent experiments.

Supplemental Figure 4

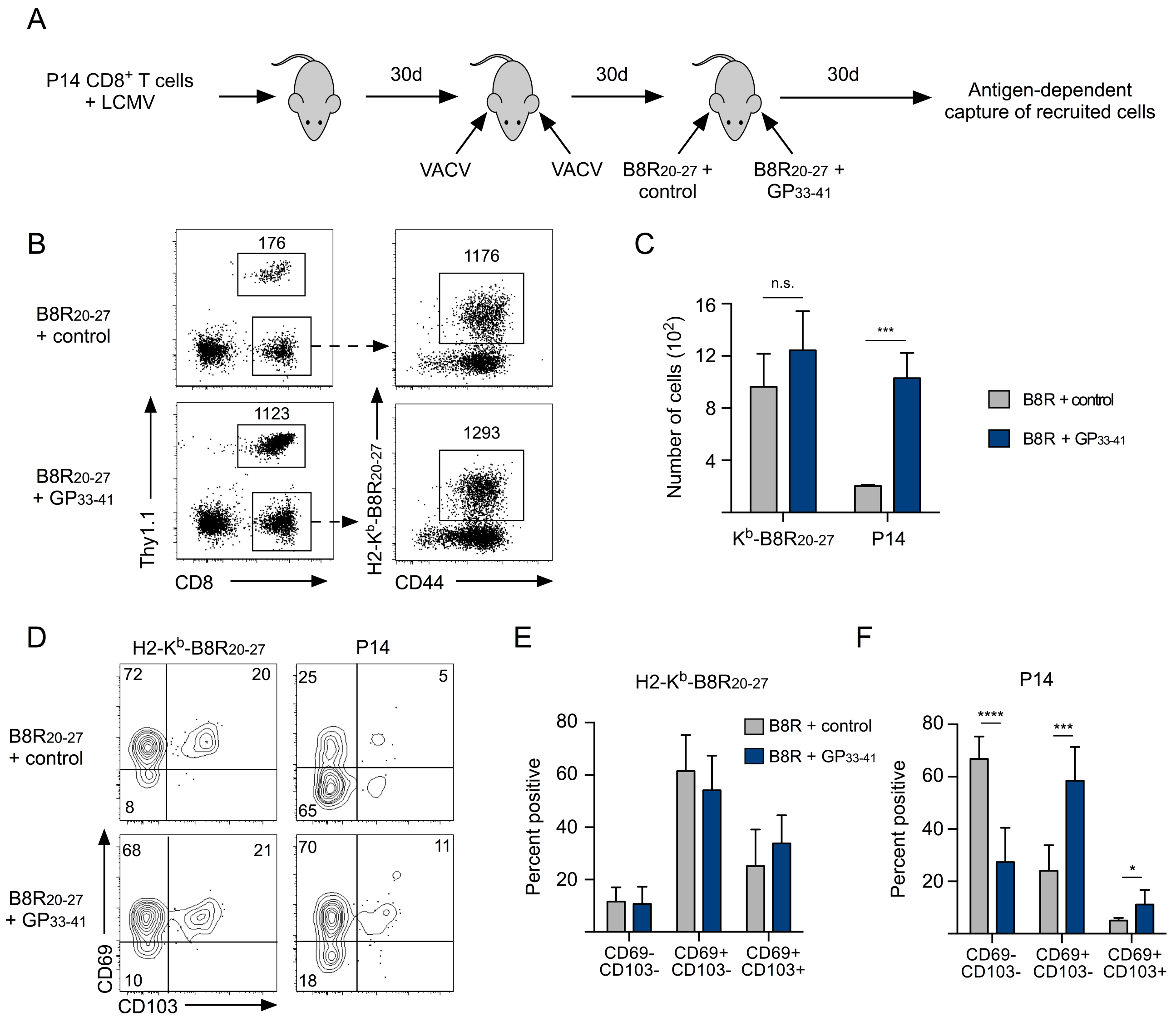

Supplemental Figure 4: Circulating memory TCR-tg CD8⁺ T cells recruited during DTH form a de novo T_{RM} CD8⁺ T cell population following local antigen recognition (Related to Figure 4). Mice received naïve P14 CD8⁺ T cells and were infected with LCMV. Mice were then infected on the left and right ear skin with VACV 30 days after LCMV infection. Ear skin was then challenged with $B8R_{20\text{-}27}$+control peptide ($OVA_{257\text{-}264}$) or $B8R_{20\text{-}27}$ + $GP_{33\text{-}41}$ three times and T_{RM} cells were analyzed 30 days after the final peptide challenge (as in Fig. 4E). (A) Experimental design. (B) Representative flow cytometry plots depicting the number of Thy1.1⁺ P14 CD8⁺ T cells and B8R-specific CD8⁺ T cells. (C) Quantification of B. (D) Representative flow cytometry plots of CD103 and CD69 expression by P14 and B8R-specific CD8⁺ T cells identified in B. (E) Quantification of CD103 and CD69 expression by B8R-specific CD8⁺ T cells identified in D. (F) Quantification of CD103 and CD69 expression by P14 CD8⁺ T cells identified in D. Statistical significance was determined using a paired t-test. Data are represented as mean ± SEM. Data are representative of 2 independent experiments (n = 5).
